# Central role of cardiac fibroblasts in myocardial fibrosis of diabetic cardiomyopathy

**DOI:** 10.3389/fendo.2023.1162754

**Published:** 2023-03-31

**Authors:** Yanan Cheng, Yan Wang, Ruili Yin, Yongsong Xu, Lijie Zhang, Yuanyuan Zhang, Longyan Yang, Dong Zhao

**Affiliations:** Beijing Key Laboratory of Diabetes Prevention and Research, Center for Endocrine Metabolic and Immune Diseases, Beijing Luhe Hospital, Capital Medical University, Beijing, China

**Keywords:** diabetic cardiomyopathy, cardiac fibrosis, cardiac myofibroblasts, cardiac fibroblasts, imbalance of extracellular matrix synthesis and degradation, disorder of matrix metalloproteinases synthesis

## Abstract

Diabetic cardiomyopathy (DCM), a main cardiovascular complication of diabetes, can eventually develop into heart failure and affect the prognosis of patients. Myocardial fibrosis is the main factor causing ventricular wall stiffness and heart failure in DCM. Early control of myocardial fibrosis in DCM is of great significance to prevent or postpone the progression of DCM to heart failure. A growing body of evidence suggests that cardiomyocytes, immunocytes, and endothelial cells involve fibrogenic actions, however, cardiac fibroblasts, the main participants in collagen production, are situated in the most central position in cardiac fibrosis. In this review, we systematically elaborate the source and physiological role of myocardial fibroblasts in the context of DCM, and we also discuss the potential action and mechanism of cardiac fibroblasts in promoting fibrosis, so as to provide guidance for formulating strategies for prevention and treatment of cardiac fibrosis in DCM.

## 1 Introduction

Diabetes mellitus, a metabolic disease, is widely considered as a public health problem. At present, there are about 463 million people suffering from diabetes worldwide (1). Cardiovascular (CV) complications are the main causes of death in diabetic patients (2). Diabetic cardiomyopathy (DCM) is an important cardiovascular complication of diabetes mellitus, which refers to the changes of cardiac function and structure caused by diabetes, independent of any other recognized cardiac risk factors such as coronary atherosclerotic heart disease and hypertension (3, 4). The epidemiological relationship between diabetes and heart failure was confirmed 50 years ago (5). Clinical heart failure trials showed that the hospitalization rate of diabetic patients with heart failure was twice that of non-diabetic patients (6, 7). Myocardial fibrosis in DCM is a crucial factor of ventricular wall stiffness and heart failure. Therefore, early control of the progress of diabetic myocardial fibrosis is of great significance to prevent or delay the progression of DCM to heart failure.

Myocardial fibrosis, known as interstitial expansion of the myocardium caused by net accumulation of extracellular matrix (ECM) (8, 9), mainly includes three distinct forms according to the basic histopathological analysis (10). In myocardial infarction, myocardial cells that are necrotic due to ischemia are replaced by collagen-based scars, resulting in “replacement fibrosis”. With the net accumulation of ECM proteins, the endomysial and perimysial spaces were expanded, causing “interstitial fibrosis”. Also, “perivascular fibrosis”, which describes the expansion of the microvascular adventitia, is part of cardiac fibrosis (10). On the one hand, “replacement fibrosis” does not seem to occur as there is no acute loss of cardiomyocytes in DCM. However, abnormal metabolism could lead to unusual apoptosis of cardiomyocytes (11), and chronic myocardial cell injury provides a condition of “replacement fibrosis” for DCM exacerbation. This “replacement fibrosis” may be a form of self-protection of heart against chronic pathological injury in the early stage of DCM. Another manifestation of self-protection is that myofibroblasts have similar activity to macrophages, which have the function of phagocytizing apoptotic cells and secreting cytokines that suppress inflammatory responses (12, 13). On the other hand, DCM is in a persistent state of metabolic derangement (14) that leads to long-term existence of fibrotic irritants in the internal environment, increasement of proliferation and activation of fibroblasts (15), as well as transdifferentiation of other cell types into CFs (16), thereby causing interstitial and perivascular fibrosis that disrupts diastolic function and results in abnormal cardiac electrophysiological activity (17). In the late stage of DCM, myocardial cell injury and apoptosis are aggravated and replacement fibrosis is more obvious, which eventually leads to the cardiac contraction dysfunction.

Despite its severity and poor prognosis, DCM still lacks formal treatment guidelines or approved specific pharmacological drugs to treat it (18). Both in human patients and in animal models of diabetes, cardiac fibrosis is prominent characteristic of diabetic cardiomyopathy (19, 20). As the main ECM-producing cells in the heart, CFs are critically involved in all cardiac fibrotic conditions (21, 22). For these reasons, an in-depth understanding of the character of CFs in myocardial fibrosis in DCM may point the direction for formulating strategies to prevent and treat myocardial fibrosis.

## 2 Diabetic cardiomyopathy

DCM is a chronic disease characterized by metabolic disorder, often accompanied by fibrosis, myocardial cell apoptosis, local inflammation, oxidative stress, endoplasmic reticulum stress and mitochondrial dysfunction (23–25). It typically manifests initially with diastolic dysfunction, but with time may also evolve with systolic dysfunction, and eventually leads to heart failure (26). In 1972, the first clinical description of DCM was published, which showed the postmortem of four patients with diabetes who had died of heart failure in the absence of coronary artery disease, hypertension, or valvular heart disease (27). Myocardial hypertrophy and fibrosis were noted in the hearts of those patients, suggesting that the fibrosis is responsible for this result. These observations were supported by Regan in 1977 (28). Autopsies of 11 uncomplicated diabetic patients revealed that collagen accumulation occurred in the heart which was present as perivascular, intermuscular, or replacement fibrosis. Therefore, it is indicated that heart failure of DCM is related to cardiac fibrosis, and even to some extent, cardiac fibrosis is a basis of heart failure caused by DCM.

## 3 The cellular effects of cardiac fibrosis in DCM

**Figure 1:**
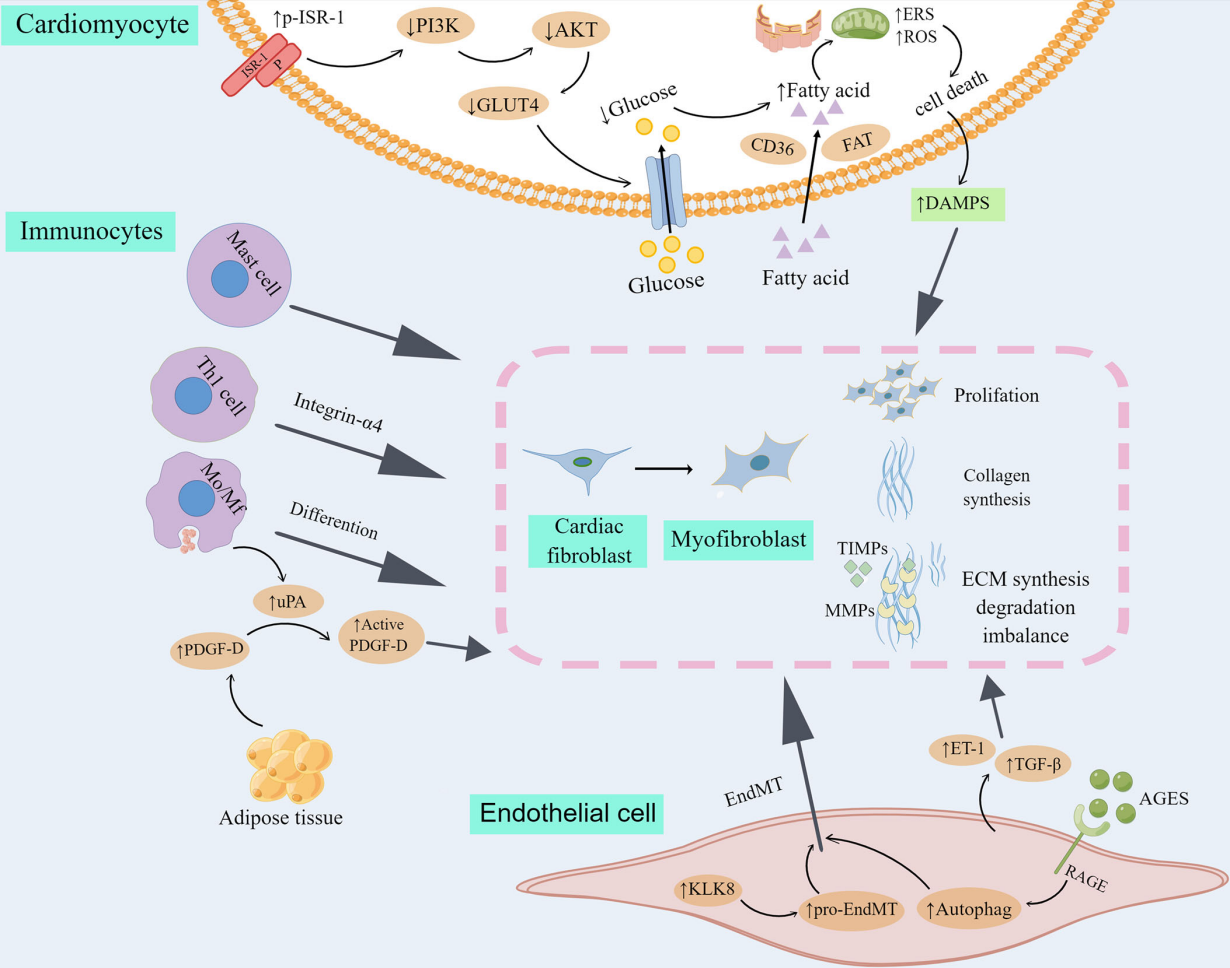
Cell types involved in myocardial fibrosis and remodeling in DCM. Transdifferentiation from cardiac fibroblast to myofibroblast is the core cellular event in cardiac fibrosis in DCM. 1) Myofibroblasts produce high level of collagen and other extracellular matrix proteins (ECM), and secrete matrix metalloproteinases (MMPs) as well as their inhibitors, tissue inhibitors of metalloproteinases (TIMPs), taking part in the progression to fibrotic remodeling. 2) Insulin resistance leads to the metabolic transformation of cardiomyocytes from glucose metabolism to fatty acid β oxidation, increased cell metabolic pressure, reactive oxygen species (ROS) and endoplasmic reticulum stress (ERS) and finally led to the death of cardiomyocytes. DAMPs released in inflammatory reaction by dead myocardial cells activate cardiac fibroblasts. 3) Immunocytes, including mast cell, Th1 cell and Mo/Mf could directly differentiate into myofibroblasts or indirectly promote cardiac fibroblast transdifferentiate into myofibroblast. 4) Endothelial cells were transformed into mesenchymal cells (EndMT), and further into myofibroblasts. In addition, fibrogenic mediators produced by endothelial cells participate in the proliferation and differentiation of myocardial fibroblasts. By Figdraw. ISR-1, insulin receptor substrate 1; P, phosphorylation; PI3K, phosphatidylinositol 3-kinase; GLUT4, glucose transporter 4; CD36, cluster of differentiation 36; FAT, fatty acid translocatase; DAMPS, danger-associated molecular patterns; Mo/Mf, monocytes/macrophages; PDGF-D, platelet-derived growth factor-D; uPA, urokinase plasminogen activator; KLK8, kallikrein-related peptidase 8; ET-1, endothelin 1; AGES, advanced glycation end products; RAGE, AGES receptor; TGF-β, transforming growth factor-beta.

Diabetes-associated cardiac fibrogenesis, including cardiac fibrosis in DCM, is a multistep and multicell process originated in cellular reaction to oxidative stress, endoplasmic reticulum stress and inflammation (26). The heart tissue is composed of cardiomyocytes and non-cardiomyocytes. After acute myocardial injury, acute death of cardiomyocytes is usually an initial event in the activation of myocardial fibrotic signaling. However, deleterious stimuli in DCM may activate pathways of fibrosis in the absence of acute cell death. Several cell types have been implicated in cardiac fibrosis and remodeling in DCM, as summarized in **Figure 1**.

### 3.1 Myofibroblasts

Myofibroblasts have been demonstrated to accumulate in myocardial interstitium under different cardiac pathological conditions (29, 30). Although the mechanism of myocardial fibrosis differ in various heart diseases, the transition from CFs to myofibroblasts is a key cellular event in cardiac fibrosis (31, 32). Myofibroblast, a kind of contractile and secretory cell type, participate in cardiac fibrotic remodeling by producing proteins of ECM (21), and by secreting matrix metalloproteinases (MMPs) as well as their inhibitors, tissue inhibitors of metalloproteinases (TIMPs). The balance between synthesis and degradation of ECM is very important in cardiac fibrosis. And the main enzyme regulating ECM degradation is MMPS, whose activity is primarily regulated by TIMPs (32–34). CFs derived from type 2 diabetes(T2DM) patients exhibited high collagen synthesis (35). And CFs isolated from diabetic heart also showed increased proliferative activity and elevated collagen and protease inhibitors expression (36, 37). *In vitro*, high glucose (HG) treatment could promote the proliferation of CFs and the collagen formation (38–40). Moreover, hyperglycemia increased the expression of transforming growth factor β (TGF-β) in CFs (41). Smads, the main downstream medium of TGF-β signaling, play important roles in the fibrosis of liver, lung, kidney and heart (42–44). Activation of TGF-β/Smads signal transduction in DCM could induce the production of MMPs and collagens, which leads to cardiac fibrosis (45, 46).

### 3.2 Cardiomyocytes

Cardiomyocytes play a critical role in myocardial fibrosis in DCM. Long-term disturbance of glucose metabolism can lead to cardiomyocyte death. First, insulin resistance leads to the increase of serine phosphorylation of insulin receptor substrate 1 (p-IRS-1), a key insulin signal factor in myocardial cells, which in turn damages the signal transduction pathway of phosphatidylinositol 3-kinase (PI3K)- protein kinase B (also known as AKT), then blocks the shift of glucose transporter 4 (GLUT4) to the cell membrane and reduces the intake of glucose in the heart (47). Moreover, fatty acid uptake regulated by fatty acid translocase (FAT) and cluster of differentiation 36 (CD36) is increased in diabetes, which causes the metabolic transformation of cardiomyocyte from glucose metabolism to fatty acid β oxidation, increased cell metabolic pressure, and ultimately cardiomyocyte death (47). In addition, up-regulation of endoplasmic reticulum stress (ERS) and reactive oxygen species (ROS) in DCM is a major cause of cardiomyocyte apoptosis (48, 49), which may eventually impair cardiac function and induce myocardial fibrosis (50). Necrotic cardiomyocytes activate immune pathways and initiate inflammatory responses. Inflammatory signals promote the infiltration of leukocytes to clear dead cardiomyocytes, while DAMPs released by the inflammatory response activate CFs, leading to the proliferation and transdifferentiation of CFs, and the deposition of a large amount of ECM to maintain the integrity of cardiac structure, but will be accompanied by the occurrence of myocardial fibrosis (51).

### 3.3 Immunocytes

Increasing evidence implicates immunocytes are involved in the regulation of cardiac fibrosis in DCM. First, the role of monocytes/macrophages (Mo/Mf) in the process of cardiac fibrosis has two sides. On the one hand, monocytes/macrophages differentiate into myofibroblasts under the action of various cytokines, producing a variety of inflammatory mediators and profibrotic factors (52). It was found that lipid metabolism disorders may lead to increased expression of full-length platelet-derived growth factor-D (PDGF-D) secreted into body fluids by adipose tissue. Urokinase plasminogen activator (uPA) produced by activated macrophages in cardiac tissue splices circulating PDGF-D into an active form that activates PI3K-AKT signaling in CFs, thereby promoting fibrosis (53, 54). The recruitment and polarization of macrophages are important processes in cardiac fibrosis (55). Traditionally, two different polarization states of macrophages are identified (29): classically activated macrophages (M1) are proinflammatory macrophages that produce proinflammatory cytokines and reactive oxygen species(ROS), and then cause the induction of inflammation and cardiac fibrosis (55, 56), whereas alternatively activated macrophages (M2) are considered as anti-inflammatory macrophages, and replacing them with M1 macrophages can inhibit cardiac fibrosis (57). On the other hand, because of their ability to devour cell debris and apoptotic cells, Mo/Mf may have anti-fibrosis effects (29, 52). Both lymphocytes and mast cells could participate in cardiac fibrosis by activating CFs. Th1 lymphocytes stimulate TGF-β to activate CFs *via* integrin-α4 (58, 59). Mast cells are capable of directly activating fibroblasts and stimulating fiber formation by histamine, tryptase, TGF-β and chymotrypsin (60).

### 3.4 Endothelial cells

Endothelial cells participation in cardiac fibrosis is suggested by the prevalence of perivascular fibrosis under the pathological conditions of infarction and pressure overload (61). Endothelial cells are the initial target of hyperglycemia damage. Sustained endothelial injury during diabetes mellitus causes endothelial-to-mesenchymal transition (EndMT), and promotes the further transformation of this endothelial cell phenotype into myofibroblasts, thus providing an additional activated fibroblasts bank and promoting cardiac fibrosis (62–64). It was found that diabetes-related EndMT and myocardial fibrosis were partly stimulated by the up-regulation of kallikrein-related peptidase 8 (KLK8). KLK8 activates platelet hemoglobin-dependent pro-EndMT signaling pathway, contributes to the evolution of EndMT and cardiac fibrosis, and accelerates the progress of cardiac dysfunction in diabetes (65). Consistently, inhibition of EndMT could reduce diabetic myocardial fibrosis (66–70), and EndMT seems to be regulated by autophagy. Endothelial autophagy deficiency induces interleukin 6 (IL-6)-dependent EndMT and cardiac fibrosis in mice with metabolic defects (71). The deletion of advanced glycation end product receptor (RAGE) has been shown to inhibit excessive autophagy and ameliorated cardiac fibrosis (72). AGES/RAGE- autophagy EndMT axis participates in the development of cardiac fibrosis, and knocking out RAGE can improve cardiac fibrosis by reducing autophagy-regulated EndMT (73). In addition, fibrogenic factors generated by endothelial cells, such as Endothelin 1 (ET-1) and TGF-β, play an important role in the pathogenesis of diabetic cardiac fibrosis (74–76). ET-1 can promote the proliferation of myocardial fibroblasts, enhance the synthesis of type I and type III collagen, resulting in the differentiation of myofibroblasts (77, 78). At the same time, ET-1 is also involved in the fibrogenic reaction of TGF-β (41, 79, 80).

Many cell types play important roles in the heart during the formation and expansion of myocardial fibrosis in DCM, and importantly, there is evidence that CFs from DCM mice and rats exhibit increased proliferation and an imbalance in ECM synthesis and degradation (**Table 1**), and this process is a central event in cardiac fibrosis in DCM.

**Table 1:** Association between diabetic cardiomyopathy (DCM) and cardiac fibroblasts (CFs).

Model of DCM	Performance of CFs	References
T1DM mice (induced by streptozotocin)	Fatty acid oxidation damage, ECM synthesis and degradation imbalance.	(81)
	Increased in numbers	(82)
	Activation	(83)
	Increased EndM	(69)
T1DM rats (induced by streptozotocin)	Induced apoptosis, inflammation and oxidative stress by activation autophagy.	(84)
	Increased autophagy	(85)
	Increased proliferation and collagen secretion.	(86, 87)
T2DM rats (induced by streptozotocin and high fat diet)	Increased oxidative stress and reactive oxygen species production.	(88)
	Increased EndMT and fibroblast activation	(89)
	Increased proliferation and collagen secretion	(90)

EndMT, endothelial-to-mesenchymal transition; T1DM, type 1 diabetes mellitus; T2DM, type 2 diabetes mellitus.

## 4 The origin and pathophysiological function of cardiac fibroblasts

### 4.1 The origin and physiological function of cardiac fibroblasts

**Figure 2:**
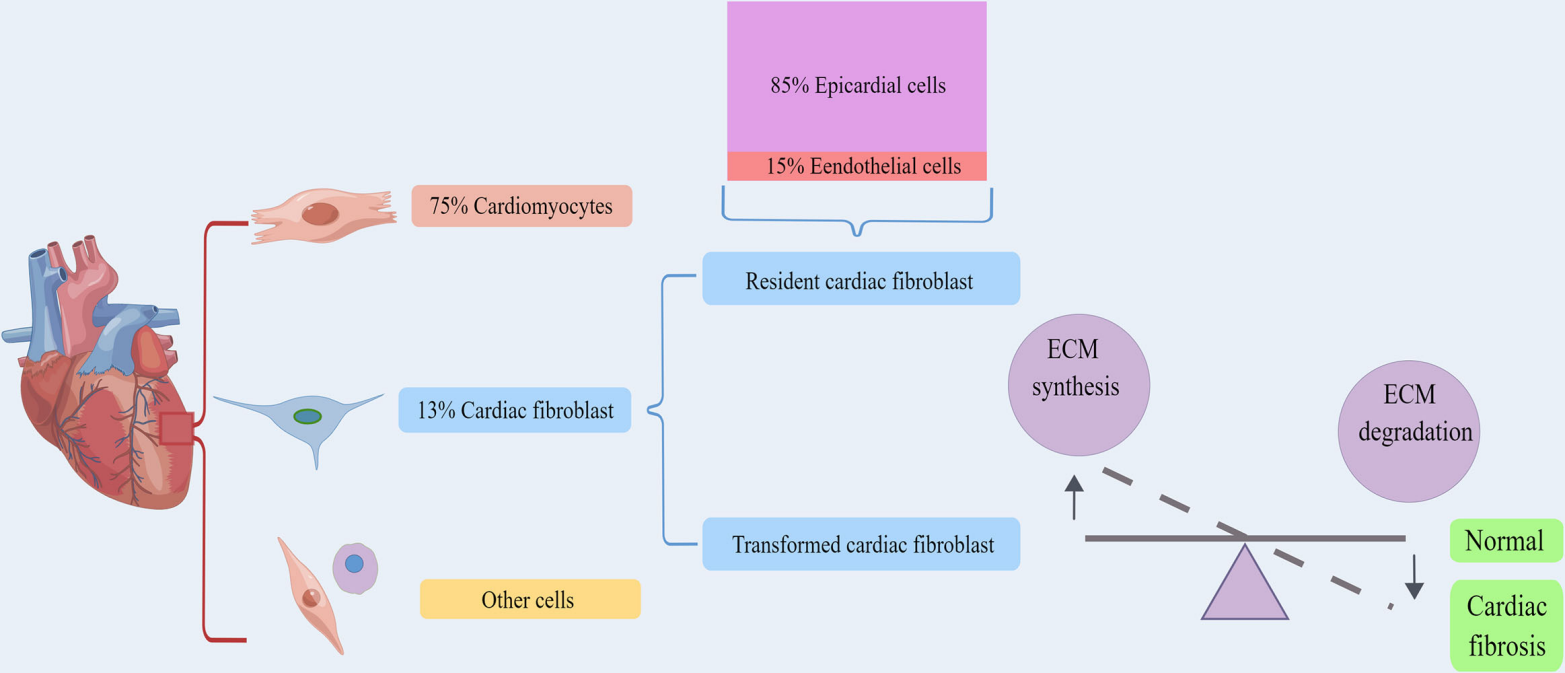
The origin and physiological function of cardiac fibroblasts. Cardiomyocytes account for about 75% of the volume of myocardium, and fibroblasts accounted for about 13%. Cardiac fibroblasts including resident cardiac fibroblast and transformed cardiac fibroblast. Approximately 85% of cardiac fibroblasts come from epicardial cells, while the other 15% are derived from endothelial cells. Both of them could serve to maintain the structural integrity of the ECM network and regulate collagen renewal. By Figdraw. ECM, extracellular matrix.

In the heart of adult mammals, cardiomyocytes account for about 75% of the volume of myocardium, and they are surrounded by the ECM network mainly composed of fibrous collagen (**Figure 2**). The main function of the cardiac ECM is to serve as the mechanical support of the heart and transmit the contraction force to ensure the normal blood pumping function of the heart (32, 91, 92). CFs are the main matrix-generating cells, forming one of the largest cell groups in the normal mammalian heart, and are usually entangled in the intima (93, 94). Methods based on histology and flow cytometry have proved that fibroblasts accounted for about 13% of mouse heart cells (95, 96). Pedigree tracing technique was used to study the origin of mouse CFs. It was found that epicardial progenitor cells differentiated into CFs and vascular smooth muscle cells during development, and endocardium differentiated into CFs through EndMT (97–104). It was estimated that approximately 85% of CFs originate from epicardial cells, while the other 15% come from endothelial cells (101). RNA analysis revealed that cells derived from epicardium and endocardium had similar expression profile (101, 104, 105), and similar multiplying activity (106). Hence, it can be inferred that there is no obvious correlation between the source and function of fibroblasts. However, it may be due to the diversity of origin and transformation that the specificity and credible marker proteins of myofibroblasts have not been reported yet (13, 31, 74, 107).

Resident CFs may serve to protect and support the constructional entirety of the ECM network and regulate collagen renewal, which is a continuous process of synthesis and decomposition of ECM proteins (108, 109). The Smad3 and platelet-derived growth factor receptor (PDGFR)-α signaling in fibroblasts are found to be vital for sustaining cell survival and cell function (110, 111). Regardless of ECM remodeling, fibroblasts play diverse functions in heart homeostasis and diseases, including providing support for multiple types of cardiac cells. In addition, CFs release paracrine factors, which may inhibit harmful inflammatory reactions and promote the survival of myocardial cells (112–116). In the early stage of diabetes, when DCM is not yet present, although the high glucose environment shocks cardiac cells, including CFs, which are in a stage of functional compensation and still able to balance ECM synthesis and degradation and yet provide a normal working microenvironment for cardiomyocytes, the heart does not exhibit obvious functional and pathological changes.

### 4.2 The role of cardiac fibroblasts in pathological conditions

In diseased heart, the cellular composition has changed dramatically. One interesting question is whether the epicardium and endocardium cells of the adult heart still have the potential to transdifferentiate into CFs. Early studies detected the presence of epithelial-mesenchymal transition (EMT) in the adult heart (117–120). In response to heart injury, CFs are activated into myofibroblasts, and endothelial cells are derived into CFs (105, 121), and macrophages can also be transdifferentiated into fibroblast-like cells (122). CFs are also derived from bone marrow hematopoietic cells (123), although the contribution of these cells to the cardiac fibroblast cluster is minimal (101, 104, 105). Cells derived from different states possess diverse expression types. Active fibroblasts have high proliferation and migration activities, and their functions include recruiting inflammatory cells, stimulating the synthesis of type I and III collagen, and determining the proliferation of fibroblasts, as well as ECM degradation mediated by MMPs, which leads to a disrupted balance between ECM synthesis and degradation and results in cardiac fibrosis (**Figure 2**) (52, 107). Some researchers have proposed four different states of fibroblasts based on the infarcted model (13, 94, 121, 122): resting fibroblasts, active fibroblasts, myofibroblasts, and matrifibrocytes. During DCM, fibroblasts stimulated by local inflammatory cytokines in the heart get the activity of proliferation and ECM production, and enter a state of ECM expansion. Activated fibroblasts can change their phenotype into matrifibrocytes. Myofibroblasts produce ECM, while matrifibrocytes express genes related to bone and cartilage remodeling (their physiological significance in the heart is unclear). Because of their location at the scar site, matrifibrocytes may be unique to acute ischemic heart injury, and active CFs may not change to this cell type in chronic heart injury such as DCM. Based on this, during the progression of DCM, the transformation of endothelial cells, epithelial cells, macrophages into cardiac fibroblasts occurs more frequently in the heart due to the stimulation of adverse factors such as local inflammatory factors, AGES, and high glucose (23–25, 124), which, together with increased activation and proliferation of cardiac fibroblasts and impaired autophagy pathways (124–126), further creates the imbalance between ECM synthesis and degradation in cardiac tissue and causes and aggravates cardiac fibrosis in DCM.

## 5 Role of cardiac fibroblasts in the development of DCM

### 5.1 Abnormal glucose metabolism affects cardiac fibroblasts

The transport of glucose in cardiac tissue is mediated by GLUT4. Insulin binding to its receptors activates insulin receptor substrate (IRS)-1/2 and downstream AKT, which stimulates the transfer of GLUT4 to cell membrane and subsequent glucose absorption (47). In patients with diabetes mellitus, the decrease of PI3K/AKT signal transduction and the high expression and translocation of GLUT4 have been detected in ventricular tissue (127). Knockout of insulin receptor reduces cardiac glucose intake, which in turn increases the generation of cardiac reactive oxygen species (ROS), triggers mitochondrial dysfunction, and increases fibrosis and heart failure (128, 129). A large number of investigations have shown that HG can induce activation, proliferation, collagen synthesis and enhanced expression of α-SMA in neonatal CFs (130–133). Sustained hyperglycemia leads to the high activation of CFs, and persuades CFs to differentiate into myofibroblasts, leading to ECM accumulation and myocardial fibrosis in cardiac tissue (36, 37, 134). *In vitro*, HG treatment increased the expression of calcium-sensitive receptor (CaSR), α-SMA, collagen I/III and MMP2/9, and enhanced the generation of autophagy and the proliferation of CFs (126). CaSR is a mediator of intracellular calcium level. It is supposed that the increase of intracellular calcium concentration could attract Smad specific E3 ubiquitin protein ligase 2 (Smurf2) expression, thus degrading SKI like proto-oncogene (SnoN) and Smad family member 7 (Smad7) proteins through ubiquitin proteasome signaling pathway (135, 136). HG and CaSR agonist significantly enhance the expression of TGF-β and p-Smad2/3, and degrade Smad7, resulting in the increase of collagen secretion in CFs (126). Therefore, continuous hyperglycemia stimulation can up-regulate CaSR expression in CFs, increase intracellular Ca^2+^ level, stimulate Smurf2 autophagy and ubiquitin proteasome, and promote collagen secretion (126, 137). In addition, HG can increase the expression of DNA methyltransferase1 (DNMT1) in CFs, cause hypermethylation of suppression of cytokine signaling 3 (SOCS3), downregulate the expression of SOCS3, and activate signal transducer and activator of transcription 3 (Stat3), thus promoting the activation of CFs and collagen deposition (138). At the same time, HG can also regulate the expression of Methyl CpG binding protein 2 (MeCP2), resulting in the methylation of target gene promoter inhibiting the expression of ras association domain family 1 isoform A (RASSF1A) and extracellular signal-regulated kinase 1/2 (ERK1/2) pathway, thus triggering cardiac fibroblasts proliferation and DCM (86). However, it was found that hyperglycemia alone could not stimulate the activation of adult CFs, pointing out that the direct influence of hyperglycemia on fibroblasts is not the main driving factor of fibrosis and remodeling in cardiac diabetic maladjustment (139). In fact, the phenotypic changes of neonatal CFs stimulated by HG *in vitro* are consistent with those *in vivo*, which indicates that it is still important to study neonatal CFs *in vitro* for DCM.

### 5.2 Abnormal lipid metabolism affects cardiac fibroblasts

In the utilization of metabolic substrates, CFs are very flexible, and relatively more dependent on fatty acid oxidation than glucose oxidation, which is similar to the integral heart (140). Obesity and insulin resistance models showed that CFs obtained enhanced myofibroblast/fibrotic gene expression and decreased reactiveness to TGF-β (141). In accordance with this, CFs isolated from db/db mice showed enhancement in collagen synthesis and decrement in TGF-β reaction (36). In response to high-fat diet (HFD), CFs have the ability to differentiate into adipocytes (142). Resistin is a hormone derived from adipocytes, which is related to obesity, insulin resistance and diabetes (143, 144). Increased resistin expression can be detected in cardiac myocytes and CFs after myocardial infarction and in neurohumoral stimulation responses (145). Studies have shown that resistin gene knockout mice fed with high fat did not exhibit relevant changes in cardiac fibrosis (146). Cardiac tissue overexpressed with resistin presented all the major markers of fibroblasts as well as the phenotypic characteristic of fibrosis, and showed augmented expression of ECM proteins including α-SMA, COL1a1, CTGF/CCN2, fibronectin, LOX, and SF2 (146). Resistin stimulates the proliferation of adult mouse CFs, activates janus kinase 2 (JAK2) by binding to toll-like receptor 4 (TLR4), and then phosphorylates Stat3, which transfers to the nucleus and activates the expression of fibroblasts target genes (146). Cell death-inducing DFFA-like effector C (CIDEC) is a vital regulator in lipid, glucose metabolism, and insulin sensitivity (147, 148). In CFs model of insulin resistance, the expression of CIDEC is increased, accompanied by nuclear translocation of CIDEC (from cytoplasm to nucleus), leading to the inhibition of AMP-activated protein kinase α (AMPK) phosphorylation and further promotes collagen synthesis (149).

### 5.3 Advanced glycation end products affect cardiac fibroblasts

Advanced glycation end products(AGES) accumulate naturally in the body, and usually exist to a lesser extent in healthy individuals (150). The accumulation of AGES occurs at a higher rate in diabetic patients (151). AGES upregulates the expression and protein level of type I collagen gene in adult rat CFs, which shows that AGES has the ability to directly promote fibrosis of CFs (152). AGES can bind to its receptor, RAGE, triggering the activation of various signal cascades, leading to downstream events, such as oxidative stress increasement, ECM reshaping and myofibroblast differentiation (153, 154). The activation of AGE/RAGE signaling pathway promotes differentiation of fibroblasts into myofibroblasts T2DM (36). The elevated AGES within diabetic collagen mediates the contraction of CFs *via* the increase of RAGE signal (155), and the increase of myofibroblast differentiation may lead to the increase of matrix contraction (156), in turn, the increased matrix contraction promotes the transformation of myofibroblasts, thereby increasing cardiac fibrosis (36, 153, 157). The migration level of diabetic CFs is higher than that of non-diabetic CFs, and along with the addition of AGES, the migration increases (158). Compared with non-diabetic collagen, higher levels of AGES were found in the tail collagen of diabetic mice (155). A 3D collagen matrix model made of tail collagen of diabetes mellitus was used to imitate the conditions *in vivo* and evaluate the changes of fibroblast function. Results showed that the increase of AGES in ECM could not only amplify the phenotypic transformation of CFs to myofibroblasts through RAGE cascade by signal transduction, but also change the cellular function and surrounding ECM, causing the increase of matrix contraction, thus accelerating the pathological remodeling of the heart (155). In addition, AGES can increase the crosslinking of matrix proteins (such as collagen) secreted by activated CFs, thus contributing to a more rigid ECM (36, 159, 160).

**Figure 3:**
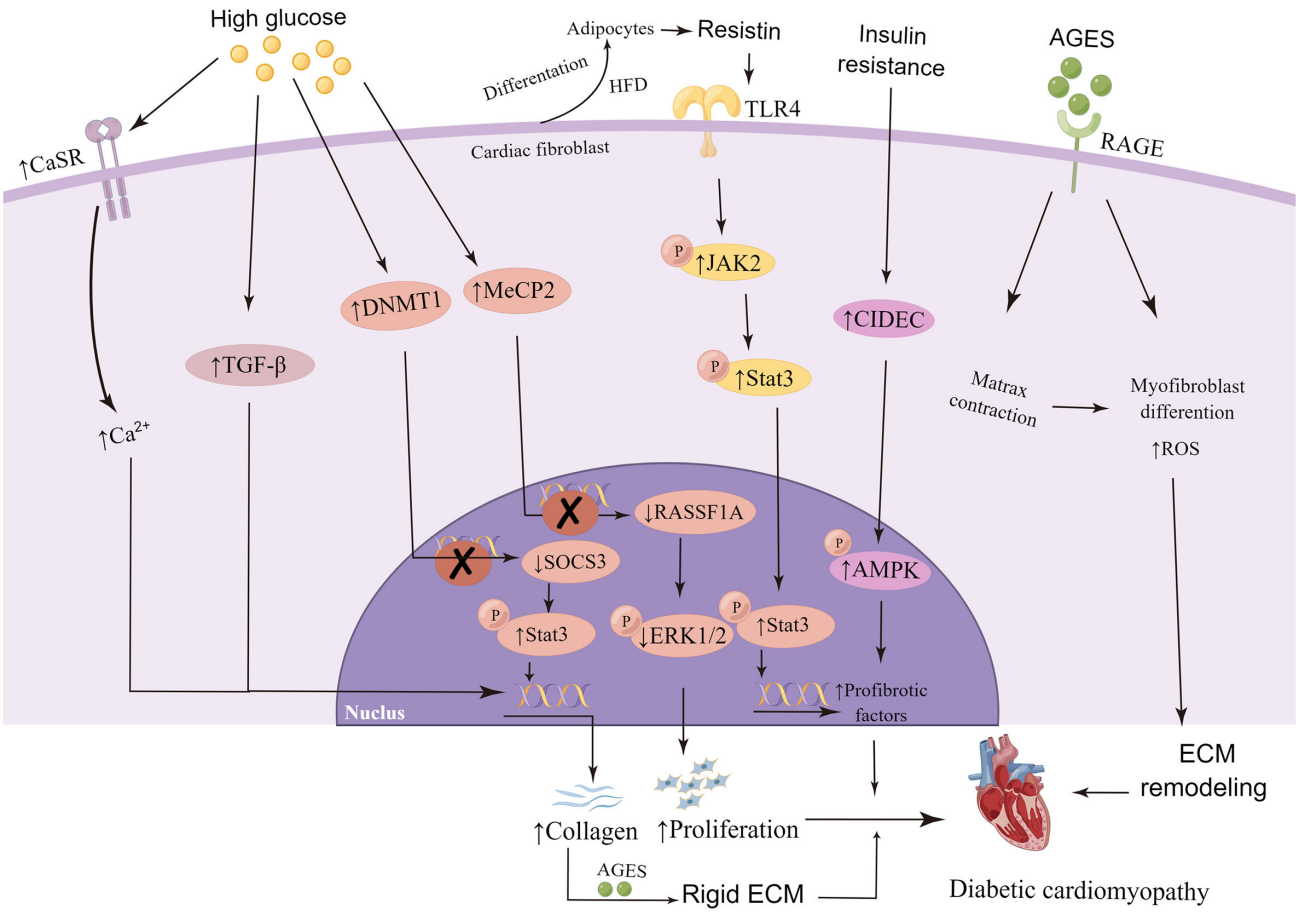
High glucose, abnormal lipid metabolism and advanced glycation end products (AGES) affects cardiac fibroblasts in the development of diabetic cardiomyopathy. Continuous hyperglycemia stimulation up-regulates the expression of calcium-sensitive receptor (CaSR), transforming growth factor-beta1 (TGF-β), DNA methyltransferase 1 (DNMT1) and Methyl CpG binding protein 2 (MeCP2) in cardiac fibroblast, and result in the increase of collagen secretion, deposition or proliferation, eventually promote the development of DCM. Resistin released by adipocytes increased expression of profibrotic factors. Accumulation of AGES bind to its receptor, RAGE, triggering the activation of various signal cascades, leading to downstream events, such as increased oxidative stress, extracellular matrix (ECM) remodeling and myofibroblast differentiation. By Figdraw. CaSR, calcium-sensitive receptor; TGF-β, transforming growth factor-beta; SOCS3, suppression of cytokine signaling 3; p-Stat3, phosphorylation of signal transducer and activator of transcription 3; RASSF1A, ras association domain family 1 isoform A; p-ERK1/2, phosphorylation of extracellular signal-regulated kinase 1/2; HFD, high-fat diet; TLR4, toll-like receptor 4; p-JAK2, phosphorylation of janus kinase 2; CIDEC, cell death-inducing DFFA-like effector C; p-AMPK, phosphorylation of AMP-activated protein kinase; ROS, reactive oxygen species.

Thus, in the early stage of diabetes, the high glucose internal environment, the abnormal lipid metabolism of cells and the cytotoxicity generated by AGES complement each other to promote the phenotypic transformation of cardiac fibroblasts, cause the secretory dysfunction of cardiac fibroblasts, trigger cardiac fibrosis, gradually aggravate with the progression of the disease, and finally cause the occurrence of adverse outcomes of diabetic cardiomyopathy (**Figure 3**).

## 6 Mechanism of myocardial fibroblasts on myocardial fibrosis in DCM

### 6.1 Typical TGF-β/Smad signaling pathway

**Figure 4:**
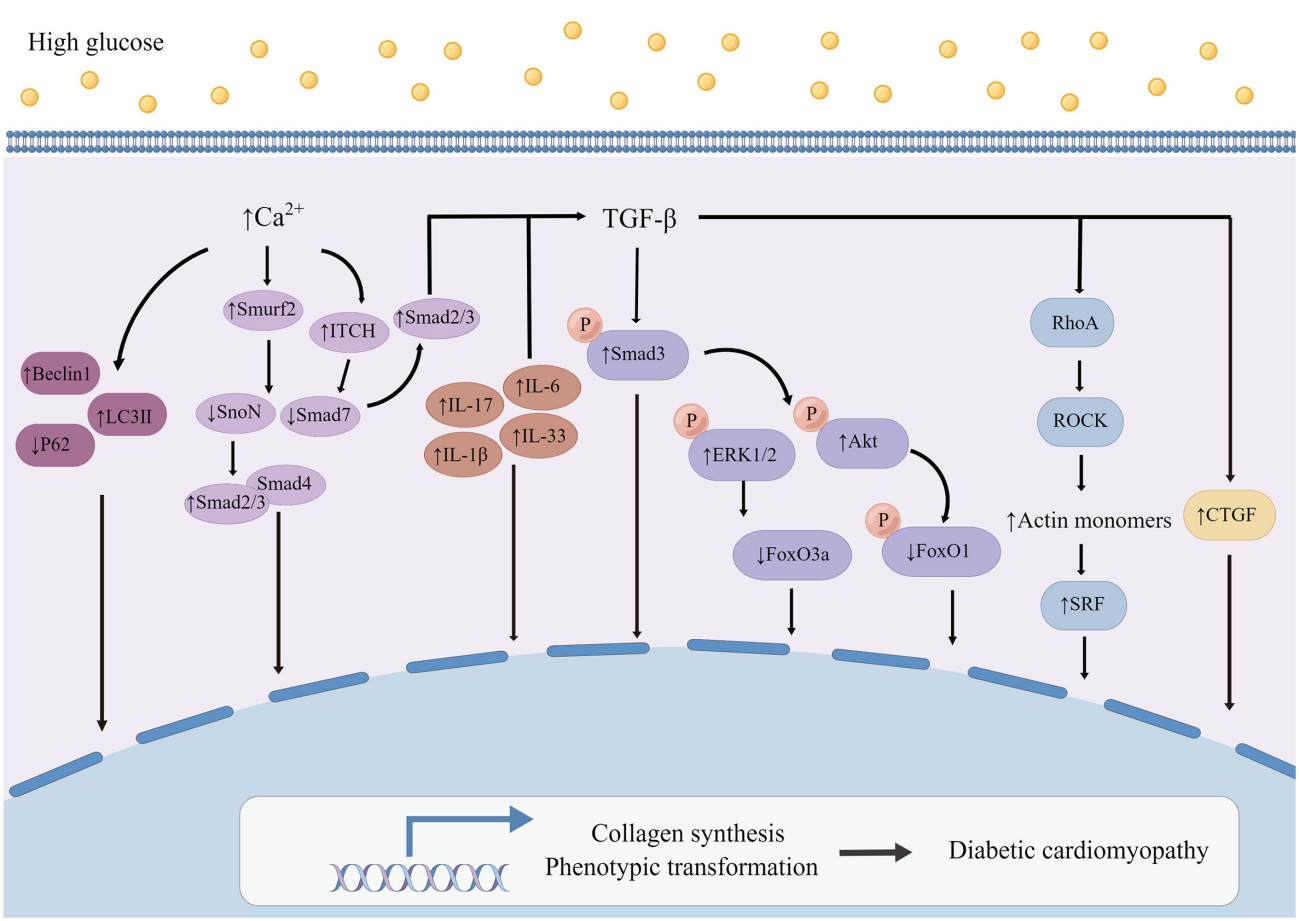
Mechanism of myocardial fibroblasts on myocardial fibrosis in diabetic cardiomyopathy. High glucose increases the expression and activity of TGF-β and causes a series of changes in signal pathways regulating the phenotypic transformation and synthesis function of CFs. The expressions of Interleukin (IL), including IL-6, IL-17, IL-1β and IL-33, were all up-regulated in myocardial fibroblasts attacked by HG, especially, IL-6 seems to up-regulate collagen gene by inducing transforming growth factor-beta 1 (TGF-β). At the same time, increased Ca^2+^ concentration regulated by CaSR could modulate the ubiquitination of Smad7 to affect DCM. By Figdraw. Smurf2, Smad specific E3 ubiquitin protein ligase 2; SnoN, SKI like proto-oncogene; Smad2/3, Smad family member 2 and Smad family member 3; Smad4, Smad family member 4; Smad7, Smad family member 7; ITCH, itchy E3 ubiquitin protein ligase; p-ERK1/2, phosphorylation of extracellular signal-regulated kinase 1/2; p-Smad3, phosphorylation of smad family member 3; p-AKT, phosphorylation of protein kinase B; p-FoxO1, phosphorylation of forkhead box O1; RhoA, ras homolog family member A; ROCK, Rho-associated coiled-coil containing kinases; SRF, serum response factor; CTGF, connective tissue growth factor.

TGF-β is a strong inducer involved in the differentiation of CFs into myofibroblasts, resulting in fibrosis (**Figure 4**) (161). Additionally, experiments *in vitro* also showed that HG can increase the expression and activity of TGF-β (162–165). Stimulated by TGF-β, CFs play a key role in the fibrosis process of cardiac ECM remodeling by synthesizing and secreting various components of ECM, such as type I and type III collagen (8). In the study of mice with selectively deleted TGF-β receptors Tgfbr1/2, Smad2, or Smad3 (which are important components for regulating TGF-β signal transduction) in CFs, it was found that regulating TGF-β signal transduction plays an important role in activating CFs (166). TGF-β stimulates the activation of typical Smad2/3 and AMPK signaling pathways (161). The specific deletion of Tgfbr1/2 and Smad3 notably reduce cardiac fibrosis resulting from transverse coarctation of aorta, and Smad3 is activated downstream of TGF-β receptor (166, 167). *In vivo* drug research found that inhibiting TGF-β/Smad2/3 pathway can inhibit the proliferation and collagen production of CFs and attenuate the degree of cardiac fibrosis (168). In recent years, there are several researches focus on how TGF-β signaling pathway regulates the phenotypic transformation and function of CFs. It was found that TGF-β activates Smad3 to regulate phosphorylation of downstream ERK1/2 and AKT, inhibits the expression of FoxO3a in CFs, and results in the increase of CFs transformation and collagen synthesis (169). This team also found that HG resulted in decreased AKT activity and AKT-mediated phosphorylation level of forkhead box O1 (FoxO1), leading to the increase of the nuclear localization and transcription activity of FoxO1, which in turn led to the phenotypic transformation of myofibroblasts and the increase of α-SMA and collagen expression (165).

### 6.2 Atypical TGF-β signaling pathway

The difference in cardiac hypertrophy responses of Tgfbr1/2 and Smad2 and Smad3 (Smad2/3) specific cardiac fibroblasts deficient mice to pressure overload stimulation indicates that atypical TGF-β signal also plays an effective role in cardiac fibroblast-mediated remodeling after chronic injury (166). It has been found that TGFβ-RhoA-ROCK pathway plays an important part in the formation of actin stress fiber, which is necessary for the differentiation of myofibroblasts (170–172). The phosphorylation of actin mediated by ROCK increases the number of actin monomers. Monomer actin loses its serum response factor (SRF) inhibitory activity. SRF and MRTF together activate the transcription of many genes, including profibrotic genes (13, 173). CFs specific deletion of ROCK2, a subtype of ROCK, significantly reduces the expression of α-SMA, decreases the expression of connective tissue growth factor (CTGF) and FGF2 in CFs, and improves cardiac function and fibrosis (174). CTGF is an important mediator in the differentiation of myofibroblasts stimulated by TGF-β (175, 176), as well as a major pathogenic factor in cardiac fibrosis (177). TGF-β has been known to increases CTGF expression in CFs (178). CTGF acts as an autocrine factor in CFs, and specific deletion of Ccn 2 (gene encoding CTGF) in CFs significantly improves cardiac function after Ang II infusion (179). Blocking the activation of TrkA, the primary receptor of CTGF, can prevent phenotype transformation and collagen production induced by HG in CFs, and CTGF knockdown shows the same result (165).

### 6.3 Interleukin

Interleukin (IL) is an important type of inflammatory cytokine. A variety of interleukin receptors are expressed in CFs, which regulate the activity of myofibroblasts (10, 22, 180, 181). It was found that the expressions of IL-6 (133), IL-17 (182), IL-1β (162) and IL-33 (183) were all up-regulated in myocardial fibroblasts attacked by HG, and loss of these inflammatory factors could reduce the collagen expression induced by HG. Under HG condition, the increased expression of IL-6 seems to up-regulate collagen gene by inducing TGF-β, which is sustained by down-regulating miR-29 by IL-6 (133). The deletion of IL-17 in diabetic mice prevents the increase of TGF-β expression (182). Therefore, interleukin mediates the ECM production of myocardial fibroblasts induced by HG (**Figure 4**), which seems to be related to the increase of TGF-β.

### 6.4 Calcium-sensitive receptor

Calcium-sensitive receptor (CaSR) belongs to the superfamily of G protein-coupled receptors, whose main function is to regulate intracellular calcium level, and it is related to many diseases, including tumor, pulmonary hypertension and cardiac infarction. It was found that HG increased CaSR expression in CFs, enhancing the autophagy and proliferation of CFs (126). Activation of CaSR leads to the increase of intracellular Ca^2+^ concentration (126). Increased intracellular calcium promots the expression of Smurf2, which has the function of degrading SnoN and Smad7 proteins through ubiquitin proteasome signaling pathway (135, 136). Smad7 can degrade Smad2/3, prevent nuclear translocation and inhibit the activation of TGF-β/Smads signaling pathway (184). SnoN belongs to the SKI proto-oncogene family, which is able to inhibit the formation of binding complex between Smad2/3 and Smad4, thus inhibiting fibrosis (185). Up-regulation of Smurf2 degrades Smad7, which leads to the weakening of the inhibitory effect of Smad7 on TGF-β/Smad2/3 signaling pathway. Meanwhile, Smurf2-siRNA significantly decreased the expression of Beclin1 and LC3II and increases the expression of p62 in CFs, which indicated that Smurf2-siRNA can down-regulate autophagy and inhibit collagen production in CFs. Studies consistent with this have also shown that CaSR activation increases Ca^2+^ concentration in myocardial fibroblasts and up-regulates the expression of itchy E3 ubiquitin protein ligase (ITCH), causing an increase in the ubiquitination of Smad7 and up-regulation of p-Smad2 and p-Smad3 expressions, thus promoting fibrosis (**Figure 4**) (186).

The investigation of these signaling pathways of cardiac fibroblasts in diabetic cardiomyopathy could provide ideas and theoretical basis for preventing or even reversing cardiac fibrosis in DCM. Future studies will still focus on understanding the alterations of cardiac fibroblast signaling pathways in diabetic cardiomyopathy with a view to finding unique targets for cardiac fibroblasts.

## 7 Future perspective

To date, Clinical attention to myocardial fibrosis in DCMis far from enough, no consensus has been reached on the best management strategy for the prevention or treatment of DCM, and there is no standard drug treatment for DCM (187). But it is encouraging that the related research is increasing year by year (188). Previous studies have shown that Irbesartan inhibits CFs proliferation and ameliorates myocardial fibrosis in T2DM rats (168). Inhibition of SGLT1 expression in CFs improves DCM cardiac fibrosis by attenuating myofibroblasts activation (90), and the SGLT1/2 inhibitor sogliflozin (zynquista) has completed phase III clinical trials (189), suggesting a novel therapeutic strategy for the treatment of DCM fibrosis. It has also been found that DAPA, the new class of antidiabetic drugs, could inhibit cardiac fibroblasts activation and EndMT to prevent cardiac fibrosis *via* TGF-β/Smads signaling (89). A recent study showed that the diabetic basic therapeutic drug Metformin is able to attenuate profibrotic gene expression in rat aortic adventitial fibroblasts (190). It can be seen that the role of CFs in DCM fibrosis is gradually valued, and fully investigating the role of CFs in myocardial fibrosis of DCM will hopefully provide power assist on targets for the treatment and prevention of myocardial fibrosis in DCM.

## 8 Conclusions

Cardiac fibrosis is one of the most critical causes of morbidity and mortality in DCM, which can lead to heart failure and increase the incidence of arrhythmia events. Although DCM has been recognized for more than 40 years, the effective prevention and treatment strategies of DCM are still elusive. Enhanced CFs proliferation, increased collagen synthesis, and disordered MMPs synthesis are the results of long-term abnormalities in glucose and lipid metabolism, which disrupt the balance between ECM synthesis and degradation in DCM, and activate multiple mechanic pathways, and ultimately increase the stiffness of ventricular wall and cardiac fibrosis. Therefore, understanding sources and changes of CFs involved in the pathogenesis of cardiac fibrosis in DCM, as well as related molecular mechanism pathways, will hopefully provide the guidance for prevention and treatment of cardiac fibrosis in DCM.

## Author contributions

YC and YW contributed equally to this work. RY, YX, LZ and YZ contributed to the manuscript revision. LY and DZ contributed to the manuscript design, discussion and revision. All authors contributed to the article and approved the submitted version.
